# Cell types or cell states? An investigation of adrenergic and mesenchymal cell phenotypes in neuroblastoma

**DOI:** 10.1016/j.isci.2024.111433

**Published:** 2024-11-19

**Authors:** Anuraag Bukkuri, Stina Andersson, Marina S. Mazariegos, Joel S. Brown, Emma U. Hammarlund, Sofie Mohlin

**Affiliations:** 1 University of Pittsburgh School of Medicine, Department of Computational and Systems Biology, Pittsburgh, PA, USA; 2 The Center for Philosophy of Science at the University of Pittsburgh, Pittsburgh, PA, USA; 3 Cancer Biology and Evolution Program and Department of Integrated Mathematical Oncology, Moffitt Cancer Center, Tampa, FL, USA; 4 Department of Experimental Sciences, Lund University, Lund, Sweden; 5 Division of Pediatrics, Department of Clinical Sciences, Lund University, Lund, Sweden; 6 Lund Stem Cell Center, Lund University, Lund, Sweden; 7 Lund University Cancer Center, Lund University, Lund, Sweden

**Keywords:** Biological sciences, Neuroscience

## Abstract

Neuroblastoma exhibits two cellular phenotypes: therapy-sensitive adrenergic (ADRN) and therapy-resistant mesenchymal (MES). To understand treatment response, it is important to elucidate how these phenotypes impact the dynamics of cancer cell populations and whether they represent distinct cell types or dynamic cell states. Here, we use an integrated experimental and mathematical modeling approach. We experimentally measure the fractions of ADRN and MES phenotypes under baseline (untreated) conditions and under repeated treatment cycles. We develop evolutionary game theoretic models predicting how the populations would respond if ADRN and MES phenotypes (1) are distinct cell types or (2) represent dynamic cell states and fit these models to the experimental data. We find that, although cells may undergo an ADRN to MES phenotypic switch under treatment, the best-fit model sees ADRN and MES as distinct cell types. Differential proliferation and survival of these two cell types, and not cell-state switching, drive therapeutic response.

## Highlights

• Repeated treatment cycles affect the fractions of neuroblastoma cell phenotypes
• Evolutionary game theory models were integrated with experimental data
• Modeling suggests the two phenotypes to be distinct cell types
• Proliferation and survival, not cell state transition, drive response to therapy

## Graphical abstract

**Figure:**
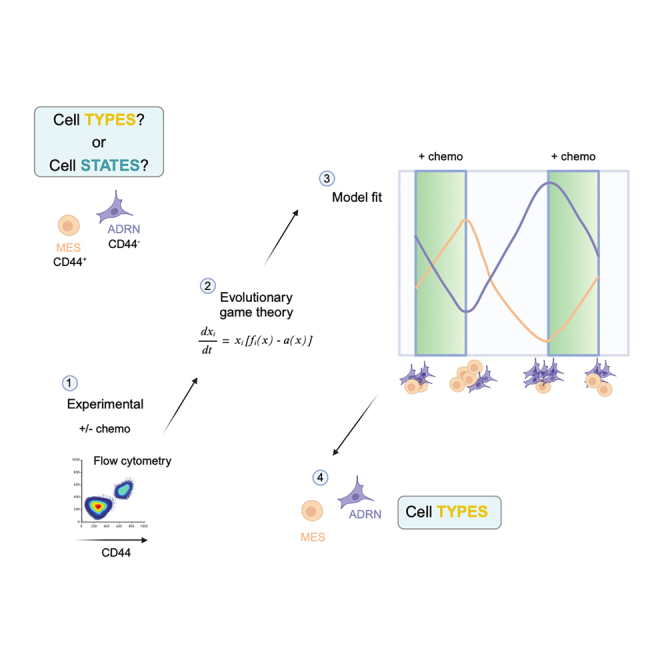

## Introduction

Neuroblastoma is a malignancy of the sympathetic nervous system. As one of the most common and deadliest pediatric cancers, neuroblastoma shows a range of clinical outcomes, from spontaneous regression to metastatic, therapy-resistant cancer with poor patient outcomes. However, the mechanisms underlying the initiation, progression, and emergence of therapeutic resistance in neuroblastoma are not fully understood. Neuroblastoma cells can develop resistance via mutations in specific pathways (primarily the Ras/mitogen-activated protein kinase signaling pathway^1^), but it has also been shown that mutation-independent phenotypic plasticity in cell state transitions may allow for adaptation to stressful environments.

In recent years, experimental studies using RNA sequencing and epigenetic profiling have found two cancer cell phenotypes in neuroblastoma with divergent gene expression profiles: adrenergic (ADRN) and mesenchymal (MES).^2^^,^^3^^,^^4^^,^^5^^,^^6^ The differentiated ADRN phenotype is more sensitive to therapy than the undifferentiated MES phenotype but comprises a higher proportion of the population under baseline conditions.^2^ How ADRN- and MES-phenotype cells respond to repeated cycles of treatment has not been investigated. Although tumors in patient most commonly present with an ADRN phenotype, using protein markers, epigenetic analyses, or bulk RNA sequencing, studies have identified MES cells in patients. A fraction of tumors even present with a preponderance of the MES phenotype.^2^^,^^7^^,^^8^^,^^9^^,^^10^ Furthermore, evidence suggests that cells may switch between these phenotypes; i.e., a cell with a MES phenotype may adopt an ADRN phenotype and vice versa.^2^^,^^8^^,^^11^ In response to chemotherapy or (anaplastic lymphoma kinase (ALK) inhibitor treatment, this phenotypic plasticity by which cells in the ADRN state facultatively switch to the MES state could play a key role in the ability of neuroblastomas to develop therapy resistance, where the fraction of cells with an adopted MES phenotype quickly expand and constitute the majority of the complete cell population in response to chemotherapy or ALK inhibitor treatment.^2^^,^^5^

However, the field lacks a consensus on the classification of these phenotypes. The ADRN and MES phenotypes are sometimes treated as separate cell types and cell states, or the distinction is ignored altogether by classifying cells as ADRN or MES like. In fact, these terms are often used interchangeably within the same study. It is critical to make a distinction between cell types and cell states, as this has important implications for the eco-evolutionary dynamics of neuroblastoma cell populations under therapy. Cell types refer to heritably distinct cellular “species” in which progeny resemble their parents; cell states refer to transient phenotypes that cells can adopt and dynamically shift between in a phenotypically plastic manner. Although some studies attempt to develop methods to delineate this difference, they have mainly taken a gene-centric approach to this problem of distinction, omitting broader implications at the population level.^12^^,^^13^

In this paper, we address the question of whether ADRN and MES phenotypes represent distinct cell types or dynamic cell states. To do this, we run experiments to measure the fraction of ADRN and MES cells in a population under untreated conditions and under repeated treatments. We then create evolutionary game theoretic mathematical models of the cell type and cell state hypotheses under untreated and treated conditions. We fit these models to our experimental data to understand whether cell type interactions or cell state transitions explain therapeutic response.

## Results

### CD44^low^ cells comprise the majority of the population under baseline conditions

**Figure 1:**
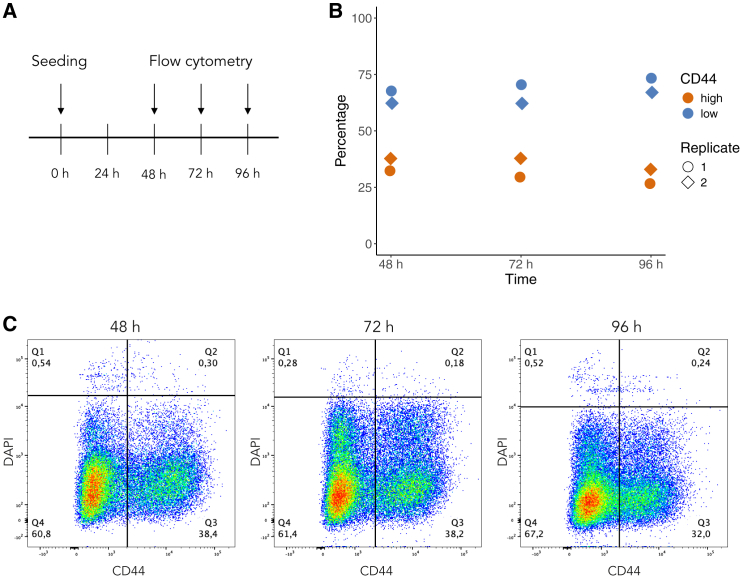
The SK-N-BE(2) cell line is dominated by CD44^low^ cells (A) Schematic image of the experimental setup. (B) Percentages of live CD44^high^ and CD44^low^ SK-N-BE(2) cells as determined by flow cytometry for two independent biological repeats. (C) Representative dot plots illustrating SK-N-BE(2) cells 48, 72, and 96 h post seeding. Q1 and Q2 represent dead cells remaining after washing, Q3 CD44^high^ cells and Q4 CD44^low^ cells.

Neuroblastoma cell lines and patient-derived tumors vary in their composition of ADRN- and MES-phenotype cells. Using neuroblastoma SK-N-BE(2) cells, we experimentally measured ADRN vs. MES dynamics under baseline conditions. Subclone SK-N-BE(2)c cells have previously been described as primarily presenting with an ADRN phenotype^2^^,^^14^; however, the fraction of each population has not previously been determined. A recent study identified CD44 as a strong and specific proxy marker for MES cells.^15^ We therefore used CD44 to distinguish ADRN (CD44^low^) and MES (CD44^high^) cells from each other. We seeded 100,000 cells and measured frequencies of ADRN and MES cells at 48, 72, and 96 h (Figure 1A). We found that the majority of SK-N-BE(2) cells presented with the ADRN phenotype (CD44^low^) at baseline (Figures 1B and 1C).

### Treatment cycles alter ADRN vs. MES proportions in the population

**Figure 2:**
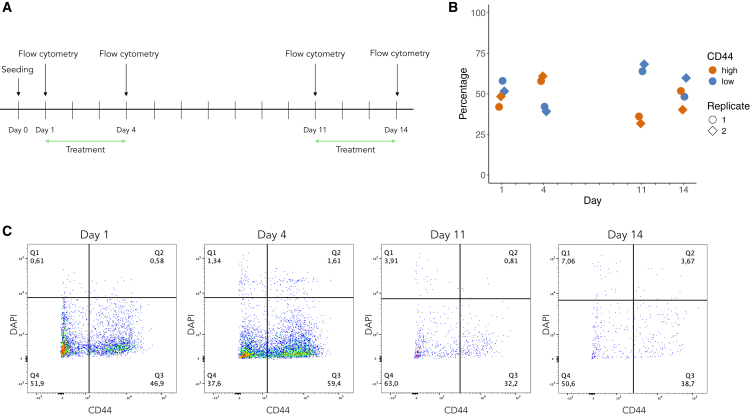
Repeated treatment with cisplatin affects CD44 expression of SK-N-BE(2) cells (A) Schematic image of the experimental setup. (B) Percentages of live CD44^high^ and CD44^low^ SK-N-BE(2) cells as determined by flow cytometry for two independent biological repeats. (C) Representative dot plots illustrating SK-N-BE(2) cells at treatment start (day 1), after 72 h of treatment (day 4), after 7 days of treatment holiday (day 11), and after a second round of 72 h of treatment (day 14). Q1 and Q2 represent dead cells remaining after washing, Q3 CD44^high^ cells and Q4 CD44^low^ cells.

Next, we examined how the frequencies of ADRN and MES cells change during prolonged treatment. We set up an experimental plan including two rounds of cisplatin chemotherapy treatment with a seven-day drug holiday in between (Figure 2A). We used the same SK-N-BE(2) neuroblastoma cells as in Figure 1 and employed CD44-based flow cytometry analysis to determine the proportion of CD44^low^ ADRN and CD44^high^ MES phenotypes in the population. Consistent with data in Figure 1B, the ADRN fraction was higher at baseline (Figures 2B and 2C). After 72 h of cisplatin treatment, the MES phenotype instead constituted 60% of the population (Figures 2B and 2C). We allowed the cells to recover, employing one week of treatment holiday, after which the ADRN phenotype resumed being the most common with nearly 70% ADRN vs. 30% MES cells (Figures 2B and 2C). Following a second round of 72 h of cisplatin treatment, the frequency of MES cells increases while the frequency of ADRN cell decreases, resulting in the two phenotypes ending up close to equilibrium (Figures 2B and 2C).

### Model construction and data fitting

The data include the proportions of CD44^high^ and CD44^low^ cells over time in untreated and treated conditions. We aimed to determine whether cell type or cell state dynamics best describe frequency dynamics and therapeutic responses. To do this, we created mathematical models of cellular dynamics for our two hypotheses: (1) ADRN and MES phenotypes as distinct cell types and (2) ADRN and MES phenotypes as cell states. The cell type hypothesis presumes that there is negligible inter-conversion between ADRN and MES cells. The cell state hypothesis assumes a stress-induced transition from the ADRN to the MES state under therapy, and the opposite switching in the absence of therapy. The cell state hypothesis may also include background phenotype-switching rates between ADRN and MES cells under baseline conditions. For both hypotheses we assume that MES cells are entirely resistant to therapy.

The modeling framework we used is that of evolutionary game theory, specifically replicator dynamics. Since our data measure the frequencies of the ADRN and MES cells instead of their abundances, this framework is more apt than classical Lotka-Volterra competition systems. The general form of the replicator equation is given by the following differential equation:

$$\frac{dx_{i}}{dt}=x_{i}\left[f_{i}\left(x\right)-\phi \left(x\right)\right]\text{,}$$

where $x_{i}$ is the frequency of phenotype i in the population, $f_{i}\left(x\right)$ captures its fitness, and $\phi \left(x\right)=\sum_{j}x_{j}f_{j}\left(x\right)$ represents the average population fitness, given as the weighted average of the fitness of all phenotypes in the population. In this way, phenotypes with a higher fitness will increase in frequency in the population at the expense of less fit phenotypes.

First, we constructed a model of cell dynamics in the untreated case. To test for state transitions between ADRN and MES phenotypes under baseline conditions, we create two models: one without background transitions between ADRN and MES phenotypes and another with these background transitions. We start with the former. Our model consists of two coupled differential equations, one to track the change in frequency of the ADRN ($x_{A}$) phenotype and one to track frequency dynamics of the MES ($x_{M}$) phenotype.

$$\frac{dx_{A}}{dt}=x_{A}\left[r\left(1-x_{A}-\alpha x_{M}\right)-\phi \left(x\right)\right]$$

$$\frac{dx_{M}}{dt}=x_{M}\left[r\left(1-{\beta x}_{A}-x_{M}\right)-\phi \left(x\right)\right]$$

The intrinsic growth rate was determined from the doubling time of SK-N-BE(2) cells of 27 h by the formula

$$r=\frac{\ln\;\left(2\right)}{27\;hours}\approx 0.026/hour$$

**Figure 3:**
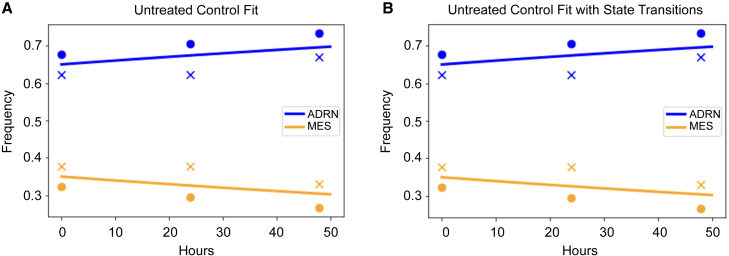
Fit of baseline model to untreated conditions Blue (orange) circles and crosses denote ADRN (MES) frequency measures from the two experimental replicates. Blue and orange lines represent the ADRN and MES model fits, respectively. (A) Fit of baseline model in untreated control. (B) Fit of baseline model in untreated control with state transitions. In accordance with data from Figures 1B and 1C, the ADRN cells have an advantage over the MES cells under baseline conditions.

To determine the interaction coefficients (α and β), we fit this model to data comprising our control data and the experimental data when treatment was off. We used least-squares minimization with the Levenberg-Marquardt algorithm (Figure 3A). When we did this, we found that α≈0.484andβ≈1.00 ($R^{2}\approx 0.994,AIC\approx -47$). There are two things to note in estimating the interaction coefficients. First, they are bounded to be no greater than 1; thus, the frequency effect of ADRN on MES hits this upper bound, leaving the frequency effect of MES on ADRN to be ≤1. In the event, α is substantially less than 1 meaning that ADRN cells have a fitness advantage over MES cells in untreated conditions, in accordance with our experimental findings in Figure 1B. Similarly, if α is bounded to less than or equal to 1, and β unbounded, then α=0.99andβ=1.26, leaving the qualitative results and outcomes unchanged.

The model with background transitions is given by

$$\frac{dx_{A}}{dt}=x_{A}\left[r\left(1-x_{A}-\alpha x_{M}\right)-\tau_{1}x_{A}+\tau_{2}x_{M}-\phi \left(x\right)\right]$$

$$\frac{dx_{M}}{dt}=x_{M}\left[r\left(1-{\beta x}_{A}-x_{M}\right)+\tau_{1}x_{A}-\tau_{2}x_{M}-\phi \left(x\right)\right]$$

To determine the competition coefficients (α and β) and background transition rates ($\tau_{1}$ and $\tau_{2}$), we again fit this model to our experimental untreated and control data using least-squares minimization with the Levenberg-Marquardt algorithm (Figure 3B). When we did this, we found virtually no change in the interaction coefficients α≈0.485andβ≈1.00 and estimates for transition rates that were negligible in size $\tau_{1}=7.36E-14\;\text{and}\;\tau_{2}=1.64E-5$ ($R^{2}\approx 0.994,AIC\approx -43$). Due to the lower AIC and lack of improvement in model fit, we conclude that background transition rates are inconsequential. For the subsequent analyses of cells under therapy, we set background transition rates to zero.

In constructing models of the populations under treatment, we start with the model for the cell type hypothesis. Since we assume that MES cells are fully resistant and that there is no switching between cell phenotypes, the model remains the same as for the untreated case, with an additional death term due to therapy, γ(t), for the ADRN cells:

$$\frac{dx_{A}}{dt}=x_{A}\left[r\left(1-x_{A}-\alpha x_{M}\right)-\gamma \left(t\right)-\phi \left(x\right)\right]$$

$$\frac{dx_{M}}{dt}=x_{M}\left[r\left(1-{\beta x}_{A}-x_{M}\right)-\phi \left(x\right)\right]$$

**Figure 4:**
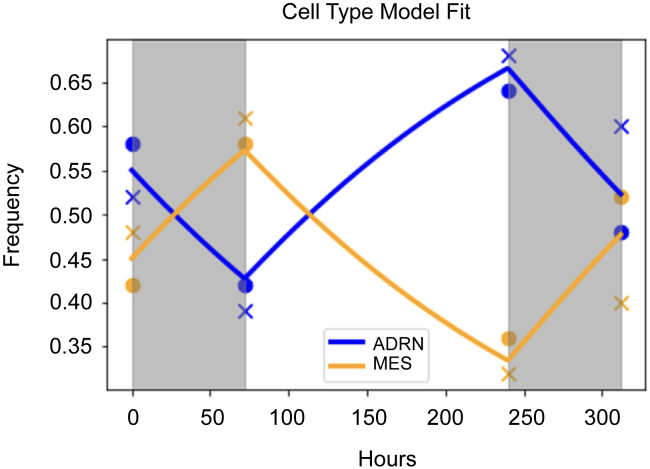
Fit of cell type model to repeated therapy cycles Blue (orange) circles and crosses denote ADRN (MES) frequency measures from the two experimental replicates. Blue and orange lines represent the ADRN and MES model fits, respectively. Regions shaded in gray denote periods of on therapy, and regions in white are periods off therapy. The model fits the data well and captures key qualitative trends: MES cells increase in frequency during periods of therapy and decrease in frequency during periods of therapy release.

Note that under periods of no therapy, γ(t)=0 and the model reduces to the untreated case. To determine the value of γ under therapy, we fit our model to our experimental data for periods of on treatment (Figure 4). We set the intrinsic growth rate and interaction coefficients to the values estimated from the untreated data: r≈0.026/hour, α≈0.484andβ≈1.00. As before, we used least-squares minimization with the Levenberg-Marquardt algorithm to estimate that γ≈0.01/day ($R^{2}\approx 0.840,AIC\approx -55$).

**Figure 5:**
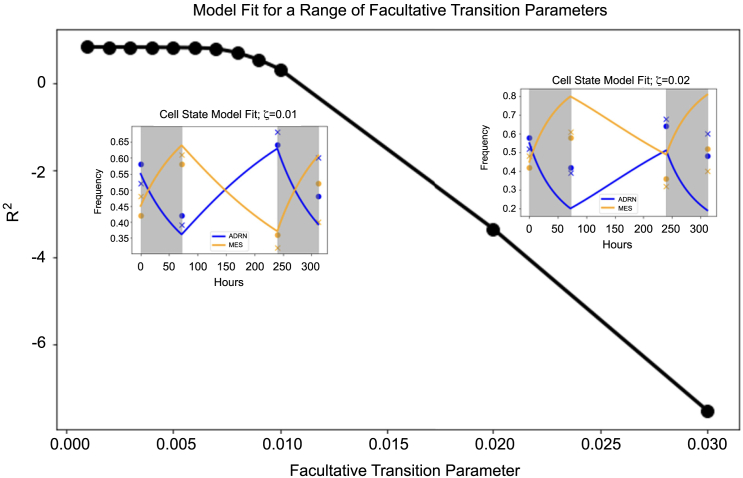
Fits of cell state model to repeated therapy cycles for a range of facultative transition parameter values Cell state transitions, if they occur at all, are likely negligible and do not contribute meaningfully to cell frequency dynamics under treatment. Forcing higher inter-conversion rates leads to poorer model fits.

Then, we modified the model for the cell state hypothesis. Once again, we assume that MES cells are fully resistant to therapy, and we allow for a facultative transition from ADRN to MES cells under therapy. Our model is then given by

$$\frac{dx_{A}}{dt}=x_{A}\left[r\left(1-x_{A}-\alpha x_{M}\right)-\gamma \left(t\right)-\zeta \left(t\right)-\phi \left(x\right)\right]$$

$$\frac{dx_{M}}{dt}=x_{M}\left[r\left(1-{\beta x}_{A}-x_{M}\right)-\phi \left(x\right)\right]+\zeta \left(t\right)x_{A}$$

where ζ is the rate at which ADRN cells switch to MES cells under therapy. Under periods of no therapy, γ(t)=ζ(t)=0, and the model reduces to the untreated case. To determine the value of γ and ζ under therapy, we fit this model to our experimental data when on treatment (Figure 5). We used least-squares minimization with the Levenberg-Marquardt algorithm to find that γ≈0.01/day and $\zeta =3.15\times 10^{-4}/day$ ($R^{2}\approx 0.841,AIC\approx -52$). This suggests that cell state transitions contribute negligibly to the frequency dynamics under therapy. Additionally, as this model has a higher Akaike information criterion (AIC) than the cell type model, it is a poorer model for capturing the relevant cell dynamics. To further explore this finding, we fixed ζ for a range of parameter values between 0 and 0.02 and performed model fits for γ. As expected, forcing higher rates of cell state transitions leads to poorer model fits with the data. Our modeling and analyses support the hypothesis that the two phenotypes represent distinct cell types whose abundances are primarily determined by survival and proliferation, and not phenotype switching.

## Discussion

The finding in 2017^2^ that neuroblastomas are composed of two distinct phenotypes, ADRN and MES, has shaped how the field views clinical outcomes and has influenced pre-clinical experimental design and analysis. While many chemotherapeutics and targeted therapies used for neuroblastoma patients are successful in the initial phase, the treatment often fails eventually. In order to overcome this issue, it is critical to understand the ecological and evolutionary dynamics of tumor populations with ADRN and MES phenotypes, particularly under therapy. In line with previously published data, we experimentally show here that neuroblastoma cell populations treated with chemotherapy *in vitro* shift from an ADRN phenotype-dominated composition to an MES phenotype. However, whether such a shift is primarily driven by phenotype switching between cell states or ecological interactions between cell types was unknown.

We used our generated experimental cell population data to test whether cell type interactions or cell state transitions drive response to therapy. We created evolutionary game-theoretical models for each hypothesis and fit them to our experimental data. These results showed that cell state transitions whether on or off therapy are likely negligibly small. Thus, frequency-dependent interactions between the cell types, as evidenced by the interaction coefficients, drive the frequency dynamics whether on or off therapy.

In general, distinct cell types display slower evolutionary dynamics in a cell population, than do phenotypic switching. Such features suggest that cell types are more responsive to adaptive therapy, whereas phenotypic switching would respond better to continuous therapy.^16^ That our data suggest that ADRN and MES phenotypes are not cell states could mean that the cancer cells cannot access rapid demographic transitions as a source of evolutionary rescue. These results suggest that neuroblastoma may be a prime candidate for adaptive therapy where therapy is stopped before the ADRN cells have been eliminated and restarted when the frequency of ADRN reaches a threshold.

### Limitations of the study

The experimental part of this study is limited to *in vitro* experiments in one cell line. Although our experiments corroborate with previously published data, our results should be validated in additional cell lines and in an *in vivo* setting. The experiments and models presented here do not account for the evolution of resistance in ADRN cells; future work could expose the population to several more rounds of therapy to probe whether evolution of resistance is relevant on clinically realistic timescales.

## Resource availability

### Lead contact

Requests for further information and resources should be directed to and will be fulfilled by the lead contact, Sofie Mohlin (sofie.mohlin@med.lu.se).

### Materials availiability

This study did not generate new unique reagents.

### Data and code availability

• This paper does not report original code.
• All data reported in this paper will be shared by the lead contact upon request.
• Any additional information required to reanalyze the data reported in this paper is available from the lead contact upon request.

## Acknowledgments

This study was funded by Längmanska kulturfonden, the 10.13039/501100001725Royal Swedish Academy of Sciences, G.S. Magnuson Foundation, the 10.13039/100023581National Science Foundation Graduate Research Fellowship Program, the 10.13039/501100002794Swedish Cancer Society, the Swedish Childhood Cancer Fund, The Crafoord Foundation, the Ollie and Elof Ericsson Foundation, the 10.13039/501100006285Magnus Bergvall Foundation, the Hans von Kantzow Foundation, and the 10.13039/501100005753Royal Physiographic Society in Lund. The authors would like to thank Anna Hammarberg and the FACS Core Facilities at MultiPark and Lund Stem Cell Center for technical expertise.

## Author contributions

A.B. and S.M. conceptualized the article. A.B. and J.S.B. developed the models and ran the simulations. A.B., S.A., M.S.M., and S.M. designed the experiments. S.A. and M.S.M. performed experiments and analyzed the experimental data. E.U.H. contributed with resources. A.B., S.A., and S.M. wrote the original draft of the paper. All authors edited and approved the final version of this article.

## Declaration of interests

The authors declare no competing interests.

## STAR★Methods

### Key resources table

**Table:** 

REAGENT or RESOURCE	SOURCE	IDENTIFIER
**Antibodies**		

Human/Mouse CD44 Alexa Fluor® 647-conjugated Antibody	R&D Systems	Cat# FAB6127R; RRID: AB_3651395
Rat IgG2B Alexa Fluor® 647-conjugated Isotype Control	R&D Systems	Cat# IC013R; RRID: AB_3271567
DAPI	Invitrogen	D3571

**Experimental models: Cell lines**		

SK-N-BE(2)	ATCC (2022)	Cat# CRL-2271; RRID: CVCL_0528

### Experimental model and study participant details

The neuroblastoma cell line SK-N-BE(2) (acquired from and authenticated by ATCC, 2022) was cultured in MEM supplemented with 10% fetal bovine serum, 100 units penicillin and 10 μg/mL streptomycin. Cells were kept at 37°C, 21% O_2_ and 5% CO_2_ in a humidified incubator and dissociated with trypsin. Cells were tested for mycoplasma at least trimonthly (Eurofins Genomics).

### Method details

#### Cell counting

Cells were stained with Trypan blue and live cells were counted in two technical replicates with a TC20 Automated Cell Counter (Bio-Rad).

#### Flow cytometry

100,000 cells were seeded to 35 mm wells and were either treated with 5 μM cisplatin (reconstituted in PBS with 140 mmol/L NaCl) after 24 h or kept as untreated control. Cells were harvested with trypsin, washed once in PBS and stained with antibodies (Table 1) in 100 μL FACS buffer (PBS, 0.5% BSA, 4mM EDTA) at 4°C avoiding light. Cells were then washed again in PBS and FACS buffer, resuspended in FACS buffer with 1:3000 DAPI and subjected to flow cytometry in BD LSRII or BD LSR Fortessa. Compensation controls, FMO controls and isotype controls were included in each run and samples were run in three technical replicates.

**Table 1:** List of antibodies

Antibody	Species	Dilution	Source	Product #
Human/mouse CD44 Alexa Fluor 647-conjugated antibody	rat	1:50	R&D Systems	FAB6127R
Rat IgG2B Alexa Fluor 647-conjugated isotype control	rat	1:50	R&D Systems	IC013R

#### Data analysis

Flow cytometry data were analyzed with FlowJo v10. Cell debris and doublets were excluded by gating SSC-A against FSC-A followed by FSC-A against FSC-W. Gating had to be adapted to each biological replicate as the autofluorescence varied between them.
